# Crystal structure of 1-(1,3-benzo­thia­zol-2-yl)-3-(4-bromo­benzo­yl)thio­urea

**DOI:** 10.1107/S2056989024004742

**Published:** 2024-05-31

**Authors:** Salif Sow, Mariama Thiam, Felix Odame, Elhadj Ibrahima Thiam, Ousmane Diouf, Javier Ellena, Mohamed Gaye, Zenixole Tshentu

**Affiliations:** a Département de Chimie, Faculté des Sciences et Techniques, Université Cheikh Anta Diop, Dakar, Senegal; b Department of Chemistry, Nelson Mandela University, Port Elizabeth, South Africa; c Departamento de Química - Facultad de Ciencias Naturales y Exactas, Universidad del Valle, Apartado 25360, Santiago de Cali, Colombia; d Instituto de Física de São Carlos, IFSC, Universidade de São Paulo, USP, São Carlos, SP, Brazil; University of Kentucky, USA

**Keywords:** crystal structure, 4-bromo­benzoyl­chloride, 2-benzo­thia­zole, potassium cyanate, thio­urea

## Abstract

The chemical reaction of 4-bromo­benzoyl­chloride and 2-amino­thia­zole in the presence of potassium thio­cyanate yielded a white solid formulated as C_15_H_10_BrN_3_OS_2_, which consists of 4-bromo­benzamido and 2-benzo­thia­zolyl moieties connected by a thio­urea group. The 4-bromo­benzamido and 2-benzo­thia­zolyl moieties are in a *trans* conformtion (sometimes also called *s*-trans due to the single bond) with respect to the N—C bond. The dihedral angle between the mean planes of the 4-bromo­phenyl and the 2-benzo­thia­zolyl units is 10.45 (11)°. The thio­urea moiety, —C—NH—C(=S) —NH— fragment forms a dihedral angle of 8.64 (12)° with the 4-bromo­phenyl ring and is almost coplanar with the 2-benzo­thia­zolyl moiety, with a dihedral angle of 1.94 (11)°. The mol­ecular structure is stabilized by intra­molecular N—H⋯O hydrogen bonds, resulting in the formation of an *S*(6) ring. In the crystal, pairs of adjacent mol­ecules inter­act *via* inter­molecular hydrogen bonds of type C—H⋯N, C—H⋯S and N—H⋯S, resulting in mol­ecular layers parallel to the *ac* plane.

## Abstract 2

A thio­urea derivative with two dissimilar functional groups was prepared and characterized. In the crystal, pairs of adjacent mol­ecules inter­act *via* inter­molecular hydrogen bonds of type C—H⋯N, C—H⋯S and N—H⋯S, resulting in mol­ecular layers parallel to the *ac* plane.

## 1. Chemical context

Benzimidazole is a heterocycle widely used in the development of therapeutic mol­ecules. Several drugs are being developed around the world and researchers continue to be inter­ested in benzimidazole derivatives and their applications (Awadh, 2023 ▸; Dhanamjayulu *et al.*, 2023 ▸; Mavvaji & Akkoc, 2024 ▸; Bandaru *et al.*, 2023 ▸). Benzimidazole derivatives with anti­cancer (Abbade *et al.*, 2024 ▸), anti­histamine (Wang *et al.*, 2012 ▸), anti­viral (Mahurkar *et al.*, 2023 ▸), anti­microbial (Bhoi *et al.*, 2023 ▸), anti­tuberculous (Kalalbandi *et al.*, 2014 ▸), anti­diabetic (Saeedian Moghadam *et al.*, 2023 ▸), anti-inflammatory (Nagesh *et al.*, 2022 ▸), anti­oxidant (Patagar *et al.*, 2023 ▸) and anti­fungal (Çevik *et al.*, 2022 ▸) properties have been reported in the literature. Thio­urea has inter­esting chemical properties, which have made it possible to develop several applications (AbdElgawad *et al.*, 2023 ▸; Fiaz *et al.*, 2024 ▸; Huang *et al.*, 2023 ▸; Eshkil *et al.*, 2017 ▸). Its high reactivity has made it possible to synthesize a very large number of derivatives with analgesic (Lee *et al.*, 2002 ▸), anti­cancer (Pingaew *et al.*, 2022 ▸), anti­microbial (Madasani *et al.*, 2023 ▸), and anti­diabetic (Faidallah *et al.*, 2011 ▸) properties. The combination of thio­urea and benzimidazole made it possible to generate new mol­ecules with properties better than those of derivatives of the two uncombined mol­ecules (Ganesh *et al.*, 2015 ▸; Harrouche *et al.*, 2016 ▸; Shang *et al.*, 2023 ▸). Mol­ecules derived from benzimidazole-thio­urea presenting potent anti­proliferative activity, compared to reference drugs, have been synthesized (Ullah *et al.*, 2022 ▸; Siddig *et al.*, 2021 ▸). It is in this context that thio­urea derivatives are the subject of particular inter­est for researchers seeking to develop mol­ecules containing one or more metal ions to improve the properties of these compounds (Muhammed *et al.*, 2024 ▸; Albrekht *et al.*, 2024 ▸; Nair *et al.*, 2022 ▸; Masaryk *et al.*, 2021 ▸). Complexes exhibiting biological properties are reported in the literature (Zhao *et al.*, 2024 ▸; Swaminathan *et al.*, 2024 ▸; Muhammed *et al.*, 2024 ▸; Albrekht *et al.*, 2024 ▸). For several years, our research group has been developing compounds containing the thio­urea moiety (Faye, Gaye *et al.*, 2022 ▸; Faye, Mbow *et al.*, 2022 ▸; Thiam *et al.*, 2008 ▸; Samb *et al.*, 2019 ▸). In this work, we report the synthesis and characterization of a mol­ecule containing both thio­urea and benzimidazole moieties. 
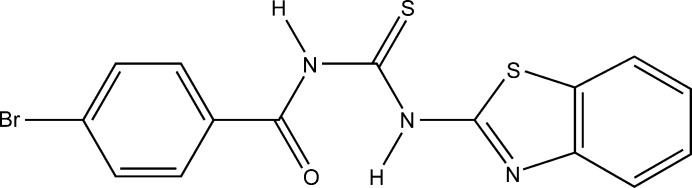


## 2. Structural commentary

**Figure 1:**
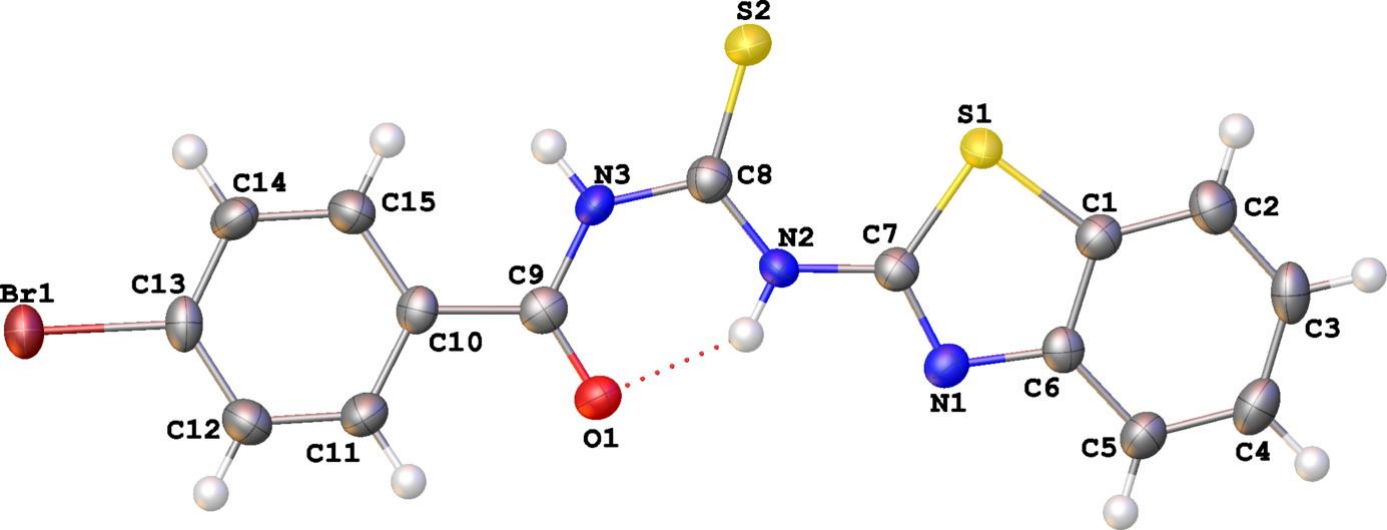
A view of the title compound, showing the atom-numbering scheme. Displacement ellipsoids are plotted at the 30% probability level.

The X-ray structure determination revealed that the title compound crystallizes in the monoclinic space group *P*2_1_/*n* with one mol­ecule in the asymmetric unit. The mol­ecular geometry is illustrated in Fig. 1 ▸. The S1—C1 [1.745 (2) Å] and the S1—C7 [1.751 (2) Å] distances indicate that these correspond to single bonds. The S2—C8 [1.663 (2) Å] and the O1—C9 [1.220 (2) Å] and N1—C7 [1.291 (3) Å] distances indicate that these correspond to double bonds and are comparable to those observed for 1,2-bis­(*N*′-benzoyl­thio­ureido)benzene [1.6574 (18) Å for C—S, 1.219 (2) Å and 1.224 (3) Å for C—O; Thiam *et al.*, 2008 ▸]. The N1—C7 [1.291 (3) Å] distance indicates double-bond character, similar to the corresponding bond length in (*Z*)-2-[(*E*)-2-(1-benzo­thio­phen-3-yl­methyl­idene)hydrazin-1-yl­idene]-1,2-di­phenyl­ethanone [1.281 (3) Å; Pekdemir *et al.*, 2012 ▸]. The N1—C6 [1.392 (3) Å], N2—C7 [1.390 (3) Å], N3—C8 [1.386 (3) Å] and N3—C9 [1.383 (3) Å] distances are in the normal range observed for a single C—N bond (Samb *et al.*, 2019 ▸; Chen *et al.*, 2001 ▸). The bond angles around N2, N3 and C8 fall in the range 115.40 (17)–128.81 (17)° and are comparable to the ideal value of 120° observed for *sp* ^2^ hybridization. The phenyl ring and the benzo­thia­zole ring are essentially planar with r.m.s deviations of 0.0081 and 0.0070 Å, respectively. The thio­urea fragment (S2/N3/N2/C8/C9) is planar with a maximum deviation from its mean plane of 0.0519 (1) Å for N3. The 4-bromo­phenyl ring and the 2-benzo­thia­zolyl groups are twisted relative to each other and form a dihedral angle of 10.45 (11)°. The two rings make dihedral angles of 8.64 (12) and 1.94 (11)°, respectively, with the thio­urea fragment. The 4-bromo­benzoyl group is *trans* with respect to the thiono S atom across the N3—C8 bond. The 2-benzo­thia­zolyl ring adopts a *cis* conformation with respect to the thiono S atom across the N2—C8 bond. The mol­ecule exhibits an intra­molecular N—H⋯O hydrogen bond (Table 1 ▸) between the carbonyl oxygen atom and the thio­amide hydrogen atom, which forms an *S*(6) ring. This phenomenon is regularly noted in the case of carbonoylurea and benzoyl thio­urea (Sow *et al.*, 2009 ▸; Woei Hung & Kassim, 2010 ▸) derivatives.

## 3. Supra­molecular features

**Figure 2:**
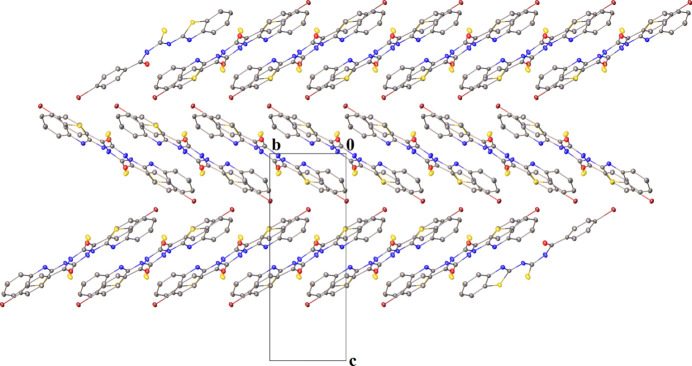
Partial packing view along the *a* axis, H atoms are omitted for clarity.

In the crystal, the mol­ecules are linked into chains that are connected by inter­molecular hydrogen bonds of type C—H⋯N, C—H⋯S, and N—H⋯S (Table 1 ▸), forming mol­ecular layers running parallel to the *ac* plane. Inter­molecular N—H⋯S and C—H⋯N hydrogen bonds further link the mol­ecules, forming a zigzag chain through 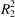 (8) rings. The inter­molecular C—H⋯S hydrogen bond consolidates the structure, forming rings of type 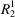 (8) (Figs. 2 ▸ and 3 ▸).

## 4. Database survey

A search of the Cambridge Structural Database (CSD version 5.44, updates of September 2023; Groom *et al.*, 2016 ▸) with the search fragment benzo­thia­zole thio­urea yielded seventeen hits. For some hits, the bromine atom is replaced by a chlorine atom (BUDZIK; Yusof *et al.*, 2009 ▸) or nitro group (HUWIM; Cui *et al.*, 2009 ▸). Other results give the same chemical formula and structure but have the bromine atom in the *ortho* or *meta* position on the benzene ring [IVEWEO (Zeng *et al.*, 2017 ▸) and SURGOE (Odame *et al.*, 2020 ▸)]. Coordination complexes based on transition metals such as rhenium (INOXUG; Schoultz *et al.*, 2016 ▸), ruthenium (NODLUQ; Shadap *et al.* 2019 ▸) and rhodium (NODMAX; Shadap *et al.*, 2019 ▸) have organic ligands that are analogues of the reported mol­ecule.

## 5. Synthesis and crystallization

The title compound was synthesized following the procedure reported by Odame *et al.* (2020 ▸) with slight modification. The thio­urea derivative was obtained by the reaction of potassium thio­cyanate (1.9388 g, 20 mmol) with 4-bromo­benzoyl chloride (4.3892 g, 20 mmol) in 25 mL of acetone and heating under reflux for 2 h to yield the 4-bromo­benzoyl iso­thio­cyanate. To the above solution was added a solution of 2-amino­benzo­thia­zole (3 g, 20 mmol) in 25 mL of acetone. The resulting mixture was heated overnight. The solvent was removed by evaporation and the crude product was recrystallized in methanol. Yield 77%, m.p. 504 K. Analysis calculated for C_15_H_10_BrN_3_OS_2_: C, 45.92; H, 2.57; N, 10.71; S, 16.35. Found: C, 45.90; H, 2.55; N, 10.69; S, 16.32. FTIR: (ν, cm^−1^): 3075 (N—H), 3015 (N—H), 1675 (C=O), 1563 (C=C), 1546 (C=C), 1451 (C—N), 1439 (C—N).

## 6. Refinement

Crystal data, data collection and structure refinement details are summarized in Table 2 ▸. H atoms were geometrically optimized (C—H = 0.95 Å, N—H = 0.88 Å) and refined as riding on their carriers with *U*_iso_(H) = 1.2*U*_eq_(C,N).

## Supplementary Material

Crystal structure: contains datablock(s) I. DOI: 10.1107/S2056989024004742/pk2706sup1.cif

Structure factors: contains datablock(s) I. DOI: 10.1107/S2056989024004742/pk2706Isup2.hkl

Supporting information file. DOI: 10.1107/S2056989024004742/pk2706Isup3.cml

CCDC reference: 2341267

Additional supporting information:  crystallographic information; 3D view; checkCIF report

## Figures and Tables

**Figure 3:**
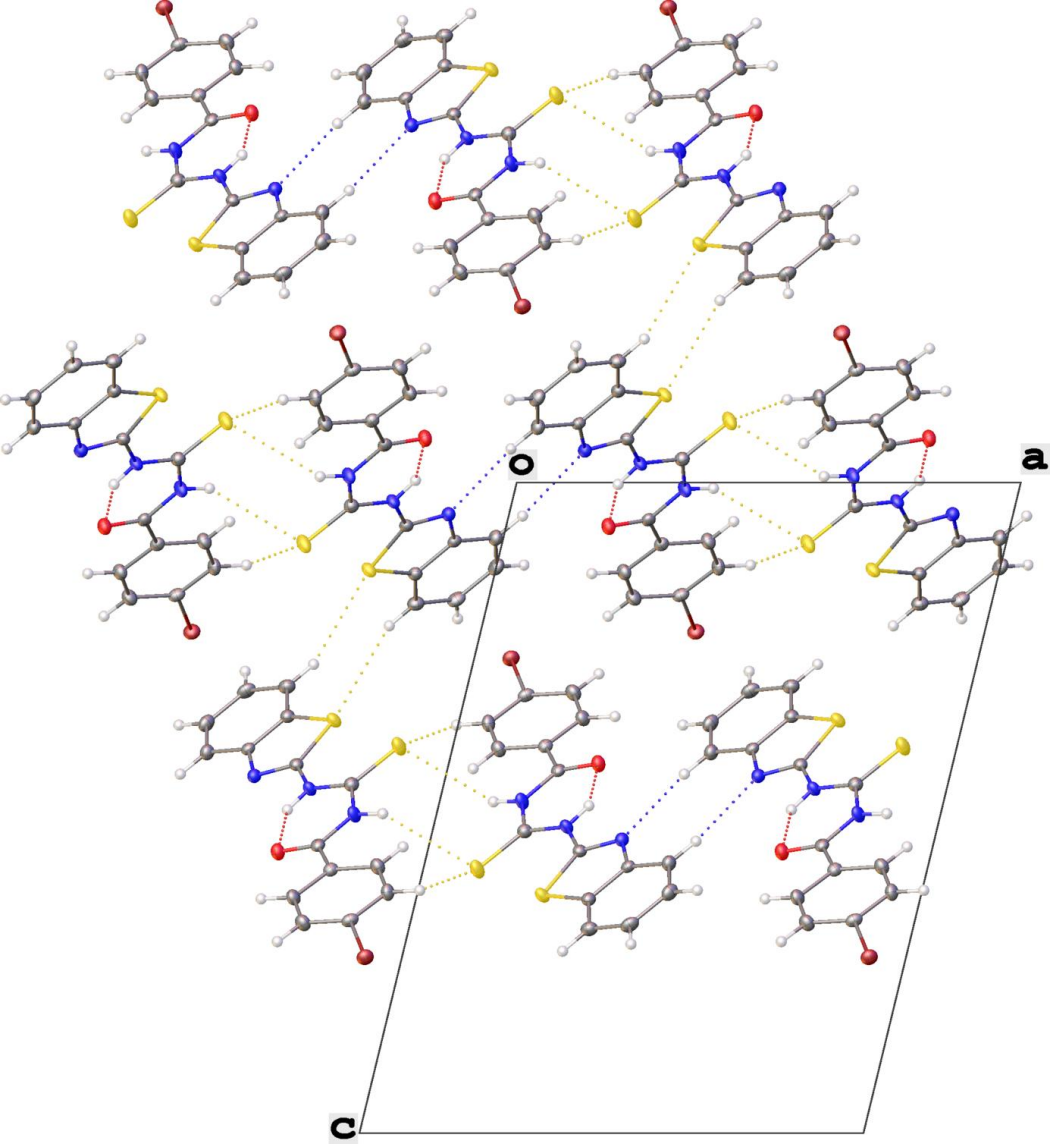
Partial packing view down the *b* axis showing the formation of 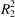 (8) graph-set motifs. Hydrogen bonds are drawn as dashed lines.

**Table 1:** Hydrogen-bond geometry (Å, °)

*D*—H⋯*A*	*D*—H	H⋯*A*	*D*⋯*A*	*D*—H⋯*A*
N3—H3⋯S2^i^	0.88	2.96	3.6102 (19)	132
N2—H2⋯O1	0.88	1.90	2.633 (2)	139
C14—H14⋯S2^ii^	0.95	2.95	3.779 (2)	146
C2—H2*A*⋯S1^iii^	0.95	3.00	3.908 (2)	161
C5—H5⋯N1^iv^	0.95	2.68	3.604 (3)	165

Symmetry codes: (i) 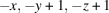 ; (ii) 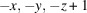 ; (iii) 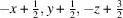 ; (iv) 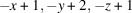 .

**Table 2:** Experimental details

Crystal data	
Chemical formula	C_15_H_10_BrN_3_OS_2_
*M*_r_	392.29
Crystal system, space group	Monoclinic, *P*2_1_/*n*
Temperature (K)	100
*a*, *b*, *c* (Å)	13.5009 (5), 6.4130 (2), 17.9147 (7)
β (°)	103.606 (4)
*V* (Å^3^)	1507.54 (10)
*Z*	4
Radiation type	Mo *K*α
μ (mm^−1^)	3.01
Crystal size (mm)	0.10 × 0.06 × 0.06

Data collection	
Diffractometer	XtaLAB Synergy, Dualflex, HyPix
Absorption correction	Gaussian (*CrysAlis PRO*; Rigaku OD, 2022 ▸)
*T*_min_, *T*_max_	0.804, 0.986
No. of measured, independent and observed [*I* > 2σ(*I*)] reflections	18792, 3074, 2581
*R*_int_	0.041
(sin θ/λ)_max_ (Å^−1^)	0.625

Refinement	
*R*[*F* ^2^ > 2σ(*F* ^2^)], *wR*(*F* ^2^), *S*	0.024, 0.057, 1.03
No. of reflections	3074
No. of parameters	199
H-atom treatment	H-atom parameters constrained
Δρ_max_, Δρ_min_ (e Å^−3^)	0.35, −0.30

Computer programs: *CrysAlis PRO* (Rigaku OD, 2022 ▸), *SHELXT* (Sheldrick, 2015*a* ▸), *SHELXL2018/3* (Sheldrick, 2015*b* ▸) and *OLEX2* (Dolomanov *et al.*, 2009 ▸).

## supplementary crystallographic information

### Crystal data

**Table:**

C_15_H_10_BrN_3_OS_2_	*F*(000) = 784
*M**_r_* = 392.29	*D*_x_ = 1.728 Mg m^−^^3^
Monoclinic, *P*2_1_/*n*	Mo *K*α radiation, λ = 0.71073 Å
*a* = 13.5009 (5) Å	Cell parameters from 9123 reflections
*b* = 6.4130 (2) Å	θ = 3.1–33.9°
*c* = 17.9147 (7) Å	µ = 3.01 mm^−^^1^
β = 103.606 (4)°	*T* = 100 K
*V* = 1507.54 (10) Å^3^	Block, light colourless
*Z* = 4	0.10 × 0.06 × 0.06 mm

### Data collection

**Table:**

XtaLAB Synergy, Dualflex, HyPix diffractometer	2581 reflections with *I* > 2σ(*I*)
Detector resolution: 10.0000 pixels mm^-1^	*R*_int_ = 0.041
ω scans	θ_max_ = 26.4°, θ_min_ = 3.1°
Absorption correction: gaussian (CrysAlisPro; Rigaku OD, 2022)	*h* = −16→16
*T*_min_ = 0.804, *T*_max_ = 0.986	*k* = −8→8
18792 measured reflections	*l* = −22→21
3074 independent reflections	

### Refinement

**Table:**

Refinement on *F*^2^	Primary atom site location: dual
Least-squares matrix: full	Hydrogen site location: inferred from neighbouring sites
*R*[*F*^2^ > 2σ(*F*^2^)] = 0.024	H-atom parameters constrained
*wR*(*F*^2^) = 0.057	*w* = 1/[σ^2^(*F*_o_^2^) + (0.0191*P*)^2^ + 1.3729*P*] where *P* = (*F*_o_^2^ + 2*F*_c_^2^)/3
*S* = 1.03	(Δ/σ)_max_ = 0.001
3074 reflections	Δρ_max_ = 0.35 e Å^−^^3^
199 parameters	Δρ_min_ = −0.30 e Å^−^^3^
0 restraints	

### Special details

**Table:**

Geometry. All esds (except the esd in the dihedral angle between two l.s. planes) are estimated using the full covariance matrix. The cell esds are taken into account individually in the estimation of esds in distances, angles and torsion angles; correlations between esds in cell parameters are only used when they are defined by crystal symmetry. An approximate (isotropic) treatment of cell esds is used for estimating esds involving l.s. planes.

### Fractional atomic coordinates and isotropic or equivalent isotropic displacement parameters (Å^2^)

**Table:**

	*x*	*y*	*z*	*U*_iso_*/*U*_eq_	
Br1	0.07298 (2)	−0.50717 (3)	0.27034 (2)	0.01865 (7)	
S1	0.24869 (4)	0.98772 (8)	0.63447 (3)	0.01659 (12)	
S2	0.10718 (4)	0.61809 (8)	0.59446 (3)	0.02191 (13)	
O1	0.29683 (11)	0.3918 (2)	0.43225 (8)	0.0182 (3)	
N3	0.16341 (14)	0.3897 (3)	0.48987 (10)	0.0172 (4)	
H3	0.106967	0.322341	0.490909	0.021*	
N1	0.38000 (13)	0.9461 (3)	0.54899 (10)	0.0155 (4)	
N2	0.26397 (13)	0.6774 (3)	0.52923 (10)	0.0148 (4)	
H2	0.298111	0.627829	0.496902	0.018*	
C10	0.18323 (16)	0.1035 (3)	0.40497 (12)	0.0145 (4)	
C11	0.24401 (17)	0.0071 (3)	0.36228 (12)	0.0183 (4)	
H11	0.307296	0.068130	0.359855	0.022*	
C8	0.18301 (16)	0.5657 (3)	0.53592 (12)	0.0167 (4)	
C9	0.22053 (16)	0.3057 (3)	0.44251 (12)	0.0152 (4)	
C13	0.12063 (17)	−0.2631 (3)	0.32704 (12)	0.0162 (4)	
C6	0.40504 (15)	1.1298 (3)	0.59049 (11)	0.0139 (4)	
C4	0.49993 (17)	1.4411 (3)	0.62991 (13)	0.0208 (5)	
H4	0.553913	1.533414	0.626970	0.025*	
C12	0.21305 (17)	−0.1773 (3)	0.32321 (13)	0.0199 (5)	
H12	0.254784	−0.243387	0.294303	0.024*	
C14	0.05956 (16)	−0.1731 (3)	0.37025 (13)	0.0178 (4)	
H14	−0.003263	−0.235813	0.372947	0.021*	
C3	0.43652 (17)	1.4872 (3)	0.67971 (13)	0.0200 (5)	
H3A	0.448160	1.611081	0.709576	0.024*	
C2	0.35797 (17)	1.3570 (3)	0.68620 (12)	0.0196 (5)	
H2A	0.315825	1.387679	0.720416	0.023*	
C1	0.34260 (16)	1.1782 (3)	0.64059 (12)	0.0158 (4)	
C7	0.30166 (16)	0.8612 (3)	0.56641 (12)	0.0144 (4)	
C15	0.09165 (17)	0.0100 (3)	0.40956 (13)	0.0185 (4)	
H15	0.050806	0.072576	0.439944	0.022*	
C5	0.48474 (16)	1.2633 (3)	0.58523 (12)	0.0179 (4)	
H5	0.527660	1.232504	0.551582	0.021*	

### Atomic displacement parameters (Å^2^)

**Table:**

	*U*^11^	*U*^22^	*U*^33^	*U*^12^	*U*^13^	*U*^23^
Br1	0.02144 (12)	0.01489 (11)	0.01875 (12)	−0.00221 (8)	0.00297 (8)	−0.00376 (8)
S1	0.0190 (3)	0.0164 (2)	0.0168 (3)	−0.0037 (2)	0.0090 (2)	−0.0033 (2)
S2	0.0203 (3)	0.0227 (3)	0.0263 (3)	−0.0068 (2)	0.0127 (2)	−0.0073 (2)
O1	0.0163 (8)	0.0172 (7)	0.0223 (8)	−0.0017 (6)	0.0071 (6)	−0.0016 (6)
N3	0.0164 (9)	0.0160 (9)	0.0212 (10)	−0.0064 (7)	0.0087 (8)	−0.0048 (7)
N1	0.0149 (9)	0.0163 (8)	0.0154 (9)	−0.0014 (7)	0.0036 (7)	0.0004 (7)
N2	0.0159 (9)	0.0147 (8)	0.0158 (9)	−0.0021 (7)	0.0074 (7)	−0.0038 (7)
C10	0.0162 (11)	0.0115 (10)	0.0150 (11)	−0.0002 (8)	0.0020 (9)	0.0018 (8)
C11	0.0174 (11)	0.0187 (11)	0.0199 (11)	−0.0035 (9)	0.0070 (9)	−0.0006 (9)
C8	0.0173 (11)	0.0165 (10)	0.0160 (11)	−0.0008 (8)	0.0035 (9)	0.0001 (8)
C9	0.0150 (11)	0.0161 (10)	0.0136 (11)	0.0012 (8)	0.0014 (9)	0.0034 (8)
C13	0.0204 (12)	0.0114 (9)	0.0147 (11)	−0.0012 (8)	−0.0002 (9)	−0.0008 (8)
C6	0.0139 (11)	0.0141 (10)	0.0123 (10)	0.0006 (8)	0.0004 (8)	0.0003 (8)
C4	0.0182 (12)	0.0189 (11)	0.0231 (12)	−0.0050 (9)	0.0004 (9)	0.0008 (8)
C12	0.0209 (12)	0.0195 (11)	0.0210 (12)	0.0005 (9)	0.0086 (10)	−0.0024 (9)
C14	0.0147 (11)	0.0166 (10)	0.0233 (12)	−0.0020 (8)	0.0066 (9)	0.0003 (8)
C3	0.0231 (12)	0.0151 (10)	0.0185 (11)	−0.0018 (9)	−0.0016 (9)	−0.0037 (8)
C2	0.0219 (12)	0.0198 (11)	0.0161 (11)	0.0002 (9)	0.0026 (9)	−0.0021 (8)
C1	0.0158 (11)	0.0171 (10)	0.0142 (11)	−0.0009 (8)	0.0029 (9)	0.0011 (8)
C7	0.0155 (11)	0.0152 (10)	0.0124 (10)	−0.0002 (8)	0.0031 (8)	0.0013 (8)
C15	0.0187 (11)	0.0175 (10)	0.0210 (12)	−0.0002 (9)	0.0081 (9)	−0.0018 (9)
C5	0.0155 (11)	0.0184 (10)	0.0197 (11)	−0.0024 (8)	0.0037 (9)	−0.0008 (8)

### Geometric parameters (Å, º)

**Table:**

Br1—C13	1.894 (2)	C11—C12	1.388 (3)
S1—C1	1.745 (2)	C13—C12	1.380 (3)
S1—C7	1.751 (2)	C13—C14	1.383 (3)
S2—C8	1.663 (2)	C6—C1	1.403 (3)
O1—C9	1.220 (2)	C6—C5	1.396 (3)
N3—H3	0.8800	C4—H4	0.9500
N3—C8	1.386 (3)	C4—C3	1.406 (3)
N3—C9	1.383 (3)	C4—C5	1.380 (3)
N1—C6	1.392 (3)	C12—H12	0.9500
N1—C7	1.291 (3)	C14—H14	0.9500
N2—H2	0.8800	C14—C15	1.386 (3)
N2—C8	1.335 (3)	C3—H3A	0.9500
N2—C7	1.390 (3)	C3—C2	1.376 (3)
C10—C11	1.391 (3)	C2—H2A	0.9500
C10—C9	1.492 (3)	C2—C1	1.395 (3)
C10—C15	1.394 (3)	C15—H15	0.9500
C11—H11	0.9500	C5—H5	0.9500
			
C1—S1—C7	87.61 (9)	C5—C4—H4	119.6
C8—N3—H3	115.6	C5—C4—C3	120.8 (2)
C9—N3—H3	115.6	C11—C12—H12	120.6
C9—N3—C8	128.78 (18)	C13—C12—C11	118.89 (19)
C7—N1—C6	109.74 (17)	C13—C12—H12	120.6
C8—N2—H2	115.6	C13—C14—H14	120.6
C8—N2—C7	128.81 (17)	C13—C14—C15	118.80 (19)
C7—N2—H2	115.6	C15—C14—H14	120.6
C11—C10—C9	116.96 (18)	C4—C3—H3A	119.3
C11—C10—C15	119.18 (19)	C2—C3—C4	121.5 (2)
C15—C10—C9	123.85 (18)	C2—C3—H3A	119.3
C10—C11—H11	119.7	C3—C2—H2A	121.3
C12—C11—C10	120.6 (2)	C3—C2—C1	117.4 (2)
C12—C11—H11	119.7	C1—C2—H2A	121.3
N3—C8—S2	118.68 (15)	C6—C1—S1	110.00 (15)
N2—C8—S2	125.92 (16)	C2—C1—S1	128.16 (16)
N2—C8—N3	115.40 (17)	C2—C1—C6	121.83 (19)
O1—C9—N3	121.88 (19)	N1—C7—S1	117.69 (15)
O1—C9—C10	122.19 (18)	N1—C7—N2	118.04 (18)
N3—C9—C10	115.92 (18)	N2—C7—S1	124.26 (15)
C12—C13—Br1	119.98 (16)	C10—C15—H15	119.7
C12—C13—C14	121.79 (19)	C14—C15—C10	120.69 (19)
C14—C13—Br1	118.22 (16)	C14—C15—H15	119.7
N1—C6—C1	114.95 (18)	C6—C5—H5	120.7
N1—C6—C5	125.29 (18)	C4—C5—C6	118.65 (19)
C5—C6—C1	119.76 (18)	C4—C5—H5	120.7
C3—C4—H4	119.6		
			
Br1—C13—C12—C11	176.90 (16)	C12—C13—C14—C15	1.1 (3)
Br1—C13—C14—C15	−177.46 (16)	C14—C13—C12—C11	−1.7 (3)
N1—C6—C1—S1	−0.6 (2)	C3—C4—C5—C6	0.0 (3)
N1—C6—C1—C2	179.95 (19)	C3—C2—C1—S1	−178.55 (17)
N1—C6—C5—C4	179.7 (2)	C3—C2—C1—C6	0.8 (3)
C10—C11—C12—C13	0.4 (3)	C1—S1—C7—N1	0.05 (18)
C11—C10—C9—O1	5.2 (3)	C1—S1—C7—N2	−178.98 (19)
C11—C10—C9—N3	−174.74 (18)	C1—C6—C5—C4	0.0 (3)
C11—C10—C15—C14	−2.0 (3)	C7—S1—C1—C6	0.32 (16)
C8—N3—C9—O1	−4.8 (3)	C7—S1—C1—C2	179.7 (2)
C8—N3—C9—C10	175.1 (2)	C7—N1—C6—C1	0.7 (3)
C8—N2—C7—S1	1.2 (3)	C7—N1—C6—C5	−179.1 (2)
C8—N2—C7—N1	−177.8 (2)	C7—N2—C8—S2	−2.1 (3)
C9—N3—C8—S2	−173.64 (17)	C7—N2—C8—N3	178.90 (19)
C9—N3—C8—N2	5.5 (3)	C15—C10—C11—C12	1.5 (3)
C9—C10—C11—C12	−177.60 (19)	C15—C10—C9—O1	−173.8 (2)
C9—C10—C15—C14	177.0 (2)	C15—C10—C9—N3	6.2 (3)
C13—C14—C15—C10	0.7 (3)	C5—C6—C1—S1	179.10 (16)
C6—N1—C7—S1	−0.4 (2)	C5—C6—C1—C2	−0.3 (3)
C6—N1—C7—N2	178.69 (17)	C5—C4—C3—C2	0.5 (3)
C4—C3—C2—C1	−0.8 (3)		

### Hydrogen-bond geometry (Å, º)

**Table:**

*D*—H···*A*	*D*—H	H···*A*	*D*···*A*	*D*—H···*A*
N3—H3···S2^i^	0.88	2.96	3.6102 (19)	132
N2—H2···O1	0.88	1.90	2.633 (2)	139
C14—H14···S2^ii^	0.95	2.95	3.779 (2)	146
C2—H2*A*···S1^iii^	0.95	3.00	3.908 (2)	161
C5—H5···N1^iv^	0.95	2.68	3.604 (3)	165

Symmetry codes: (i) −*x*, −*y*+1, −*z*+1; (ii) −*x*, −*y*, −*z*+1; (iii) −*x*+1/2, *y*+1/2, −*z*+3/2; (iv) −*x*+1, −*y*+2, −*z*+1.
